# Socioeconomic‐ and Insurance‐Based Inequities in Oncotype DX Testing and Score‐Guided Treatment

**DOI:** 10.1002/cam4.71485

**Published:** 2025-12-21

**Authors:** Courtney P. Williams, Jessica Shuey, Joud El Dick, Nusrat Jahan, Erica Stringer‐Reasor, Andres Azuero, Gabrielle B. Rocque

**Affiliations:** ^1^ Division of General Internal Medicine & Population Science University of Alabama at Birmingham Birmingham Alabama USA; ^2^ O'Neal Comprehensive Cancer Center University of Alabama at Birmingham Birmingham Alabama USA; ^3^ Division of Hematology & Oncology University of Alabama at Birmingham Birmingham Alabama USA; ^4^ School of Nursing University of Alabama at Birmingham Birmingham Alabama USA

## Abstract

**Purpose:**

Personalized approaches to breast cancer treatment are increasingly guided by expensive, lab‐based genomic testing like Oncotype DX (ODX) Breast Recurrence Score Test. Little is known about how socioeconomic and insurance status may affect utilization of ODX testing and subsequent ODX score‐guided treatment.

**Methods:**

This retrospective cohort study included women diagnosed with early‐stage, HR+/HER2‐ breast cancer from 2011 to 2023 within the US‐based, electronic health record (EHR)‐derived, deidentified Flatiron Health Research Database. Socioeconomic status was measured by a census block‐level measure of neighborhood deprivation. Insurance status was captured at the time of diagnosis. Utilization of ODX testing was compared descriptively. Age‐stratified likelihood of adjuvant chemotherapy by neighborhood deprivation or insurance status was estimated using relative risk, predicted probabilities, and 95% confidence intervals from adjusted Poisson models.

**Results:**

Of 3814 patients eligible for ODX testing, 47% were commercially insured, and 31% lived in an impoverished neighborhood. Compared to those without, patients with an ODX test (47%, *n* = 1797) were more often white (81% vs. 74%), commercially insured (50% vs. 44%), or lived in an affluent neighborhood (72% vs. 66%). For patients aged ≤ 50 with low recurrence risk, patients who resided in affluent vs. impoverished neighborhoods had a 5% higher probability of receiving potentially inappropriate overtreatment with adjuvant chemotherapy. Of those with low/medium recurrence risk, publicly insured patients had more than double the probability of receiving adjuvant chemotherapy compared to those privately insured, suggesting potential overtreatment. For patients aged > 50 with ODX with high recurrence risk, Medicare beneficiaries had a 5% lower probability than privately insured patients and an 11% lower probability than Medicaid/Other beneficiaries of receiving recommended adjuvant chemotherapy, suggesting under treatment.

**Conclusion:**

Socioeconomic‐ and insurance‐based inequities, including both overtreatment and undertreatment, were observed in this EHR‐based cohort of women with early‐stage breast cancer eligible for ODX testing, indicating opportunities to increase care quality.

## 1 Background

Personalized approaches to breast cancer treatment are increasingly guided by expensive, lab‐based genomic testing such as the Oncotype DX (ODX) Breast Recurrence Score Test, a prognostic and predictive 21‐gene assay which both estimates risk of recurrence and predicts the benefit of adjuvant chemotherapy in women with hormone receptor‐positive (HR+), HER2‐negative, early‐stage breast cancer (eBC) [1]. Both the National Comprehensive Cancer Network and the American Society of Clinical Oncology consider ODX testing as high‐quality, guideline‐based care in women with eBC [2, 3]. Use of ODX testing has resulted in decreases in overuse of adjuvant chemotherapy, reduced patient anxiety about treatment decisions, as well as improved breast cancer‐specific and overall survival [4, 5, 6, 7]. Although guideline recommended, use of ODX is suboptimal, with registry‐based estimates ranging from 25% to 50% tested of those eligible [8, 9]. Reasons for this suboptimal use are not well understood. One study found oncologist organizational (e.g., staff responsible for ordering, reporting delays), interpersonal (e.g., colleague buy‐in), and personal (e.g., difficulty communicating test results to patients) factors influenced usage of ODX testing [10]. Furthermore, racial and ethnic inequities in the use of ODX are well documented, with Black or African American and Hispanic women with eBC less often tested using ODX than non‐Hispanic White women [9, 11, 12, 13, 14].

Individuals of lower socioeconomic status or who are uninsured or underinsured (defined as out‐of‐pocket health care costs > 10% of household income) [15] may also be less likely to receive ODX testing. A previous study of women living in California with eBC found that women with low socioeconomic status or receiving Medicaid benefits less often received ODX testing [8]. This is potentially due to its high out‐of‐pocket cost of testing, estimated at $4000 [1]. An estimated 90% of insured women are enrolled in a plan that covers ODX testing, yet insured patients may still face high out‐of‐pocket expenses for ODX testing due to substantial cost‐sharing requirements, such as high deductibles. Thus, coverage itself does not guarantee low‐cost care, which may result in socioeconomic‐ and insurance‐based inequities in ODX testing.

In addition to receipt of ODX testing itself, patient socioeconomic and insurance status could impact decisions surrounding receipt of adjuvant chemotherapy based on ODX testing results. Patients with lower socioeconomic status or who are underinsured may be less likely to receive adjuvant chemotherapy due to the associated treatment costs, even if their ODX recurrence score indicates high recurrence risk. This effect was seen in a previous state‐specific study, which found Medicaid enrollees were less likely to receive adjuvant chemotherapy regardless of their ODX score [8]. Conversely, patients with higher socioeconomic status or who are adequately insured may opt to receive potentially unnecessary adjuvant chemotherapy, putting them at higher risk of treatment‐related toxicities. However, it is unknown how socioeconomic and insurance status may affect receipt of ODX testing and ODX recurrence score‐guided adjuvant chemotherapy. This data is crucial in understanding how socioeconomic and insurance status may impact ODX recurrence score‐guided treatment strategies to reduce inequitable cancer‐related financial outcomes and optimize eBC treatment for all women. Therefore, this study examines receipt of ODX testing and ODX recurrence score‐guided adjuvant chemotherapy for patients of differing socioeconomic and insurance status.

## 2 Methods

### 2.1 Study Design and Population

This retrospective cohort study examined how socioeconomic and insurance status affect receipt of ODX testing and ODX recurrence score‐guided adjuvant chemotherapy for women diagnosed from January 1, 2011 to December 31, 2023 with stage I‐II HR + HER2‐ breast cancer in US‐based electronic health record (EHR)‐derived deidentified Flatiron Health Research Database. The Flatiron Health database is a longitudinal database, comprising de‐identified patient‐level structured and unstructured data, curated via technology‐enabled abstraction [16]. During the study period, the de‐identified data originated from approximately 280 cancer clinics (~800 sites of care) across the United States. The majority of patients in the database originate from community oncology settings; relative community/academic proportions may vary depending on the study cohort. Women eligible for our study sample were aged 18 and older, had biomarker data within 6 months of eBC diagnosis, eligible for ODX testing (node negative or ≤ 3 positive nodes, < 5 cm tumor size), and received breast cancer treatment within 6 months post‐diagnosis. Exclusion criteria included having a documented MammaPrint test prior to an ODX test date, presence of metastatic disease prior to or within 6 months post‐diagnosis, lack of follow‐up data within 3 months post‐diagnosis or 6 months post‐surgery, or had missing breast cancer diagnosis or insurance enrollment dates. This study was approved by the University of Alabama Institutional Review Board (IRB‐300010313).

### 2.2 Outcome: Adjuvant Chemotherapy

Receipt of ODX score‐guided adjuvant chemotherapy was identified and included line setting (adjuvant, locoregional, metastatic, or neoadjuvant), start date, and category of drug/regimen. Treatment with adjuvant chemotherapy within 3 months post‐surgery was identified using the line start date and setting.

### 2.3 Exposure: ODX Testing and Recurrence Score

Receipt of ODX testing was identified using documentation of an ODX test, test date, and recurrence score result. ODX recurrence scores range from 0 to 100, with higher scores indicating greater likelihood of breast cancer recurrence. Interpretation of ODX scores differs by age. For women aged ≤ 50 years, scores of 0–15 indicate low recurrence risk, 16–20 low to medium recurrence risk, 21–25 medium recurrence risk, and 26–100 high recurrence risk. For women aged > 50 years, scores of 0–25 indicate low recurrence risk and scores 26–100 indicate high recurrence risk [17]. ODX scores indicating low or low/medium recurrence risk suggests chemotherapeutic benefit will likely not outweigh risk of side effects, while scores indicating medium or high recurrence risk suggests chemotherapeutic benefit will likely outweigh risk of side effects.

### 2.4 Moderators: Socioeconomic and Insurance Status

Socioeconomic status was derived from the Yost Index [18, 19], a census block‐level measure of neighborhood deprivation comprised of seven factors used in cancer surveillance including median household income, home value, and rent, and proportions of individuals living below 150% of the poverty line, considered working class, and unemployed, and education level. The Yost Index was reported as US population‐weighted quintiles, where 1 represents the most deprived neighborhoods and 5 the least deprived neighborhoods. We further dichotomized into impoverished (quintiles 1–2) and affluent neighborhoods (quintiles 3–5) [20].

Insurance status closest to eBC diagnosis date was grouped into private (commercial plan), Medicare (fee‐for‐service, Advantage, Medicare Supplement), or Medicaid/other (patient assistance program, self‐pay, other government, other unknown payer, workers compensation). Insurance status was dichotomized (private vs. Medicare/Medicaid/other) for analyses of patients aged ≤ 50 years due to the small number of Medicare beneficiaries within that age category.

### 2.5 Covariables

Covariables included birth year (age at diagnosis), year of eBC diagnosis, race and ethnicity (Non‐Hispanic White, BIPOC), cancer stage (pathologic stages I–II), practice type (academic, community), Eastern Cooperative Oncology Group (ECOG) performance status score (0–4), year of diagnosis, and comorbidity (Klabunde‐modified Charlson comorbidity score 0 vs. ≥ 1) [21, 22]. Comorbidities with a documented ICD code 1 year pre‐ to 30 days post‐eBC diagnosis were included.

### 2.6 Statistical Analysis

Patient sociodemographic characteristics were described using means and standard deviations (SD) for continuous variables and frequencies for categorical variables. Comparisons of sociodemographic characteristics between patients with and without documented ODX testing were assessed using Cohen's d (0.2 small, 0.5 medium, 0.8 large effect) or Cramer's V (0.1 small, 0.3 medium, 0.5 large effect) effect sizes to determine the magnitude of relationships in bivariate associations [23]. Associations between having a documented ODX test and patient sociodemographic and clinical characteristics were assessed using relative risks (RR) and corresponding 95% confidence intervals (CI) from exploratory, modified Poisson models [24]. Covariables included age at diagnosis, race and ethnicity, neighborhood deprivation, insurance status, practice type, comorbidity count, cancer stage, ECOG score, and year of eBC diagnosis. For patients with a documented ODX test, associations between ODX recurrence score and receipt of adjuvant chemotherapy were assessed using similar models adjusted for the same covariables as listed above. To understand potential moderating effects of neighborhood deprivation and insurance status on receipt of adjuvant chemotherapy, interactions between risk score and insurance status (SCORE*INSURANCE) or neighborhood deprivation (SCORE*DEPRIVATION) were added to our models. Interaction effects for insurance status and neighborhood deprivation were examined in separate models. Predicted probabilities and their 95% CI were also calculated. All analyses were stratified by age ≤ 50 years vs. > 50 years due to differing interpretations of ODX scores. Sensitivity analyses dichotomized ODX scores into high (high and medium recurrence risk) vs. low (low and low/medium recurrence risk) for women ≤ 50 years old. SAS 9.4 (Cary, NC, USA) was used for all analyses.

## 3 Results

### 3.1 Sample Characteristics and Receipt of ODX Testing

**FIGURE 1:**
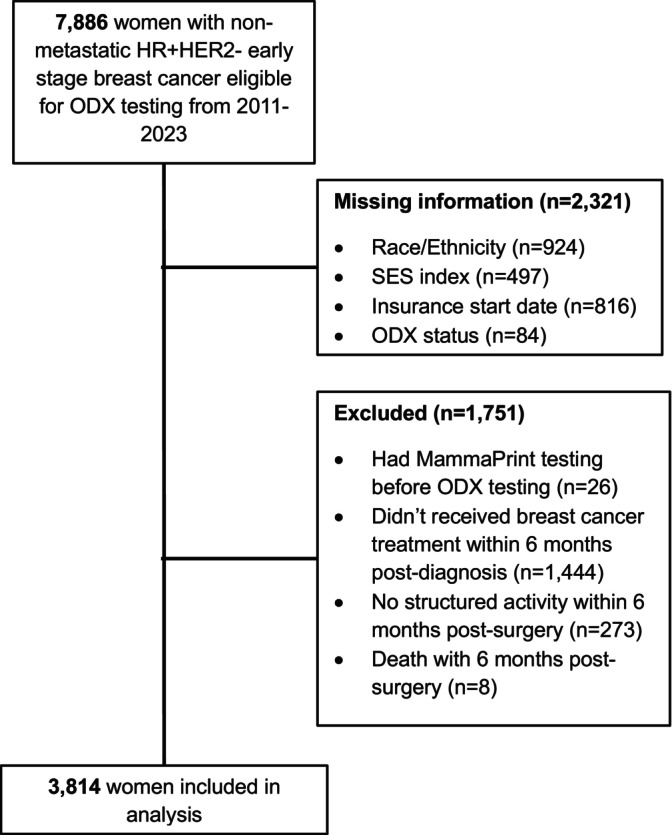
Exclusion cascade.

Within the Flatiron database, 7886 women with HR + HER2‐ non‐metastatic early‐stage breast cancer were eligible for ODX testing from 2011 to 2023. Of those, 2321 were excluded for missing data and 1725 were excluded due to having a pre‐ODX test MammaPrint test result (*n* = 26), no documented breast cancer treatment within 6 months post‐diagnosis (*n* = 1444), no post‐surgical follow‐up data (*n* = 273), or death within 180 days post‐surgery (*n* = 8; Figure 1). Compared to those included, patients excluded from our analyses were more often Medicaid/other enrollees or treated at a community practice (Table S1). A total of 3814 patients were included in the analytic sample. Patients were an average 62 years old (SD 12), 77% white, 69% had stage I cancer, 8% had at least 1 comorbidity, 47% were privately insured, and 31% lived in an impoverished neighborhood (Table 1). Almost half (47%) had a documented ODX test (*n* = 1797). Compared to those without, patients with a documented ODX test more often had an ECOG score of 0 (71% vs. 64%, V = 0.1), were white (81% vs. 74%, V = 0.1), and had stage I cancer (72% vs. 66%, V = 0.1; Table 1). While few differences in receipt of ODX testing were seen by insurance status, patients living in affluent neighborhoods had a documented ODX test more often than those living in impoverished neighborhoods (72% vs. 66%, V = 0.1; Table 1). This was also seen in exploratory models, where patients living in impoverished neighborhoods had 9% lower likelihood of having a documented ODX test than those living in affluent neighborhoods (RR 0.91, 95% CI 0.84–0.98; Table 2).

**TABLE 1:** Receipt of Oncotype DX testing by patient sociodemographic and clinical characteristics (*N* = 3814).

	Overall (*N* = 3814)	Received Oncotype DX test (*n* = 1797)	Did not receive Oncotype DX test (*n* = 2017)	Cramer's V
Age at diagnosis (mean ± SD)	62 ± 12	61 ± 10	63 ± 12	d = 0.2
≤ 50 years	669 (18)	314 (17)	355 (18)	0.002
> 50 years	3145 (82)	1483 (83)	1662 (82)	
Race/ethnicity				
Non‐Hispanic White	2942 (77)	1449 (81)	1493 (74)	0.08
BIPOC	872 (23)	348 (19)	524 (26)	
Neighborhood deprivation				
Impoverished	1181 (31)	499 (28)	682 (34)	0.07
Affluent	2633 (69)	1298 (72)	1335 (66)	
Insurance status				
Private	1792 (47)	901 (50)	891 (44)	0.06
Medicare	1365 (36)	614 (34)	751 (37)	
Medicaid/Other*	657 (17)	282 (16)	375 (19)	
Practice type				
Academic	858 (23)	421 (23)	437 (22)	0.02
Community	2956 (78)	1376 (77)	1580 (78)	
Comorbidity count				
0	3527 (92)	1676 (93)	1851 (92)	0.03
≥ 1	287 (8)	121 (7)	166 (8)	
Cancer stage				
I	2616 (69)	1287 (72)	1329 (66)	0.07
II	1033 (27)	429 (24)	604 (30)	
Unknown	165 (4)	81 (5)	84 (4)	
ECOG score				
0	2553 (67)	1270 (71)	1283 (64)	
1	726 (19)	306 (17)	420 (21)	0.11
2	98 (3)	20 (1)	78 (4)	
3	24 (1)	7 (0.4)	17 (0.8)	
4	5 (0.1)	2 (0.1)	3 (0.2)	
Unknown	408 (11)	192 (11)	216 (11)	
Diagnosis Year				
2011	169 (4)	66 (4)	103 (5)	0.07
2012	210 (6)	91 (5)	119 (6)	
2013	235 (6)	111 (6)	124 (6)	
2014	291 (8)	135 (8)	156 (8)	
2015	317 (8)	160 (9)	157 (8)	
2016	351 (9)	155 (9)	196 (10)	
2017	339 (9)	161 (9)	178 (9)	
2018	389 (10)	170 (9)	219 (11)	
2019	392 (10)	199 (11)	193 (10)	
2020	359 (9)	172 (10)	187 (9)	
2021	303 (8)	161 (9)	142 (7)	
2022	307 (8)	155 (9)	152 (8)	
2023	152 (4)	61 (3)	91 (5)	

* Other Government Program, Other Payer—Type Unknown, Workers Compensation.

**TABLE 2:** Exploratory models estimating likelihood of Oncotype DX testing by patient sociodemographic and clinical characteristics (*N* = 3814).

	Relative risk (95% confidence interval)
Age at diagnosis	0.99 (0.99–0.99)
Race/ethnicity	
Non‐Hispanic White	Ref.
BIPOC	0.82 (0.75–0.90)
Neighborhood deprivation	
Affluent	Ref.
Impoverished	0.91 (0.84–0.98)
Insurance status	
Private	Ref.
Medicare	1.03 (0.95–1.13)
Medicaid/other	0.91 (0.82–1.01)
Practice type	
Academic	Ref.
Community	1.02 (0.94–1.11)
Comorbidity count	
0	Ref.
≥ 1	0.92 (0.80–1.06)
Cancer stage	0.83 (0.76–0.91)
ECOG score	0.99 (0.98–1.01)
Cancer diagnosis year	1.01 (1.00–1.02)

### 3.2 Recurrence‐Score Guided Treatment in Patients Aged ≤ 50

**FIGURE 2:**
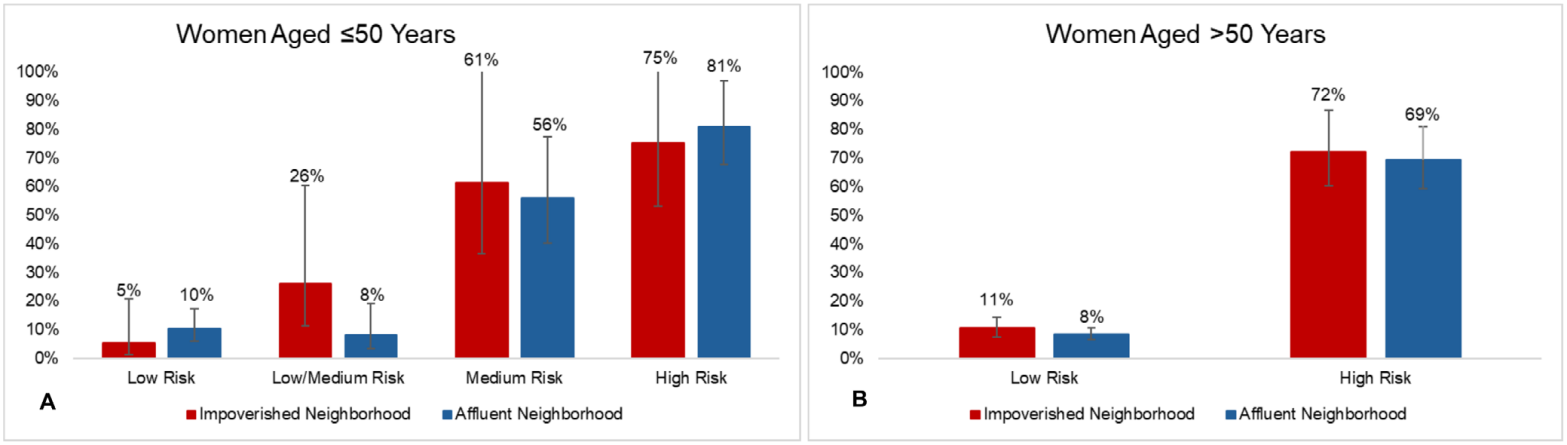
Predicted probabilities and 95% confidence intervals of receiving adjuvant chemotherapy by ODX recurrence score and neighborhood deprivation for (A) women aged ≤ 50 years (*n* = 314), and (B) women aged > 50 years who received ODX testing (*n* = 1483). *p* values < 0.05 are denoted by*.

For patients aged ≤ 50 with an ODX test (*n* = 314), 52%, 24%, 12%, and 12% had low, low/medium, medium, and high recurrence risk, respectively. Although receipt of adjuvant chemotherapy was rare for patients with low recurrence risk, patients living in affluent neighborhoods had double the probability of receiving adjuvant chemotherapy compared to those living in impoverished neighborhoods. Though this was not statistically significant, this could indicate a trend towards potential overtreatment for wealthier patients (10%, 95% CI 6%–17% vs. 5%, 95% CI 1%–21%, *p* = 0.37; Figure 2A). For those with high recurrence risk, patients living in impoverished neighborhoods had 6%, non‐significant lower probability of receiving adjuvant chemotherapy than those living in affluent neighborhoods, suggesting undertreatment for patients with fewer financial resources (75%, 95% CI 53%–106% vs. 81%, 95% CI 68%–97%, *p* = 0.91). Patients living in impoverished versus affluent neighborhoods had a non‐significant higher probability of receiving adjuvant chemotherapy in both the low/medium and medium risk categories (26% vs. 8%, *p* = 0.06; 61% vs. 56%, *p* = 0.76, respectively; Figure 2A).

**FIGURE 3:**
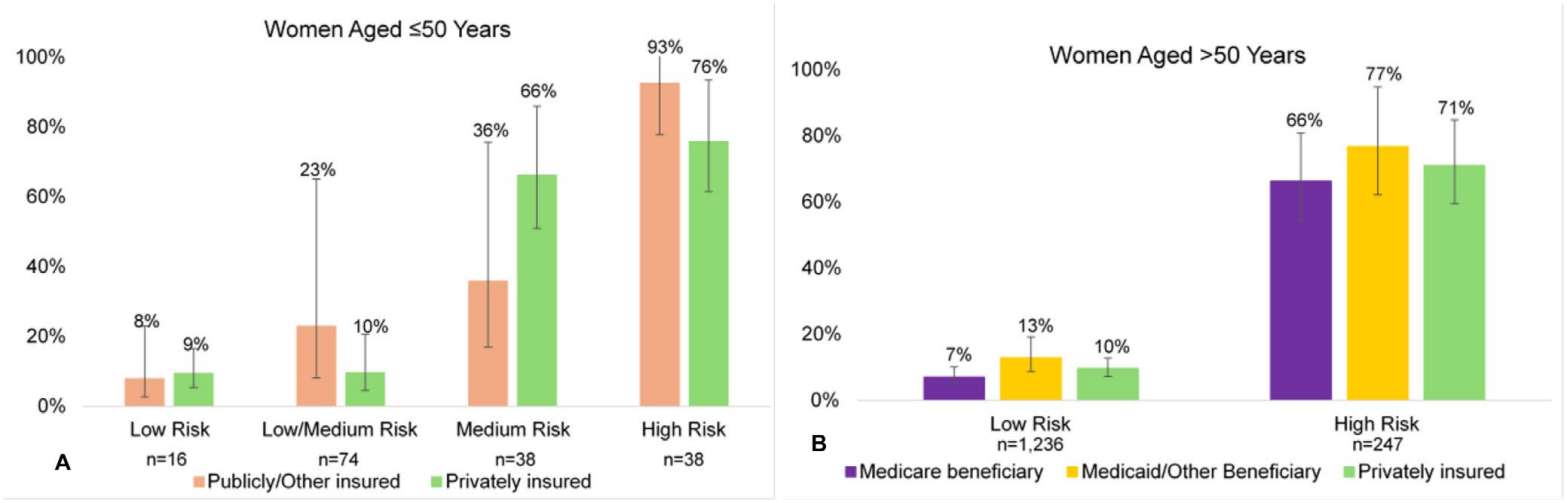
Predicted probabilities and 95% confidence intervals of receiving adjuvant chemotherapy by ODX recurrence score and insurance status for (A) women aged ≤ 50 years (*n* = 314), and (B) women aged > 50 years who received ODX testing (*n* = 1483). *p* values < 0.05 are denoted by *.

Regarding insurance, of those with low/medium recurrence risk, publicly insured patients had more than double the probability of receiving adjuvant chemotherapy compared to those privately insured (23%, 95% CI 8%–65% vs. 10%, 95% CI 4%–21%, *p* = 0.18). Though statistically non‐significant, this could suggest a trend towards potential overtreatment. Conversely, of those with medium recurrence risk, publicly insured patients had 30%, non‐significant lower probability of adjuvant chemotherapy compared to those privately insured (36%, 95% CI 17%–76% vs. 66%, 95% CI 51%–86%, *p* = 0.13; Figure 3A). Though not statistically significant, publicly insured patients at high risk for recurrence trended towards a 22% higher likelihood of receiving adjuvant chemotherapy compared to those privately insured (RR 1.22, 95% CI 0.95–1.57; Table 3). Sensitivity analyses dichotomizing ODX scores into high and low recurrence risk showed similar results (Table S2).

**TABLE 3:** Multivariable models estimating risk of Oncotype DX recurrence score‐guided adjuvant chemotherapy by age, recurrence risk, neighborhood deprivation, and insurance status.

	Women aged ≤ 50 years who received ODX testing *n* = 314	Women aged > 50 years who received ODX testing *n* = 1483
	RR (95% CI)	RR (95% CI)
Neighborhood deprivation		
Low risk, affluent neighborhood	Ref.	Ref.
Low risk, impoverished neighborhood	0.52 (0.12–2.18)	1.26 (0.87–1.82)
Low/medium risk, affluent neighborhood	Ref.	—
Low/medium risk, impoverished neighborhood	3.15 (0.95–10.48)	—
Medium risk, affluent neighborhood	Ref.	—
Medium risk, impoverished neighborhood	1.10 (0.59–2.03)	—
High risk, affluent neighborhood	Ref.	Ref.
High risk, impoverished neighborhood	0.93 (0.64–1.35)	1.04 (0.86–1.26)
Insurance status		
Low risk, privately insured	Ref.	Ref.
Low risk, publicly/other insured	0.83 (0.25–2.79)	—
Low risk, Medicare beneficiary	—	0.73 (0.47–1.13)
Low risk, Medicaid/other beneficiary	—	1.34 (0.84–2.13)
Low/medium risk, privately insured	Ref.	—
Low/medium risk, publicly/other insured	2.39 (0.66–8.69)	—
Medium risk, privately insured	Ref.	—
Medium risk, publicly/other insured	0.54 (0.25–1.19)	—
High risk, privately insured	Ref.	Ref.
High risk, publicly/other insured	1.22 (0.95–1.57)	—
High risk, Medicare beneficiary	—	0.93 (0.74–1.18)
High risk, Medicaid/other beneficiary	—	1.08 (0.88–1.33)

*Note:* All models adjusted for age at diagnosis, race and ethnicity, cancer stage, cancer diagnosis year, ECOG status, comorbidity count, and practice type.

Abbreviations: CI, confidence interval; RR, relative risk.

### 3.3 Recurrence‐Score Guided Treatment in Patients Aged > 50

For patients aged > 50 with ODX testing (*n* = 1483), 83% and 17% had low and high recurrence risk, respectively. For those at both high and low risk of recurrence, patients living in impoverished and affluent neighborhoods had similar probabilities of receiving recommended adjuvant chemotherapy (Figure 2B). Regarding insurance status, for those with high recurrence risk, Medicare beneficiaries had a 5% lower probability than privately insured patients and 11% lower probability than Medicaid/Other beneficiaries of receiving recommended adjuvant chemotherapy, suggesting undertreatment (Medicare: 66%, 95% CI 54%–81%, Private 71%, 95% CI 59%–85%, Medicaid/Other 77%, 95% CI 62%–95%; Figure 3B).

## 4 Discussion

Socioeconomic‐ and insurance‐based inequities, including both overtreatment and undertreatment, were observed in this exploratory, EHR‐based cohort of women with early‐stage breast cancer eligible for ODX testing. These inequities were more pronounced for younger women. Ensuring equitable access to personalized medicine is essential to optimizing treatment outcomes, regardless of socioeconomic status or ability to pay. Our study found that women living in affluent neighborhoods had a documented ODX test 6% more often than those living in impoverished neighborhoods, and there was little to no difference in receipt of ODX testing by insurance status. These small observed differences suggest that out‐of‐pocket costs may not be a major barrier to accessing ODX testing itself. However, we did observe potential financial barriers to receiving ODX‐guided treatment. In this era of personalized medicine, it is crucial to identify and address the economic factors that could result in treatment disparities [25].

Our descriptive results revealed no differences in receipt of ODX testing by insurance status. However, our study findings on the association between insurance status and receipt of recurrence score‐guided treatment were mixed, with both higher and lower likelihoods of treatment for both publicly and privately insured patients. This finding may stem from price sensitivities surrounding adjuvant chemotherapy, which is associated with high out‐of‐pocket costs for patients due to the cost of chemotherapeutic agents, risk of toxicities, and potential for additional healthcare utilization (e.g., emergency department visits), and the wide range of side effects and need for supportive medications. Using administrative billing claims data from 2008 to 2012, one study estimated median out‐of‐pocket costs for a course of adjuvant chemotherapy in HER2‐ patients was $3381, with 25% of patients paying more than $4712, and 10% of patients paying more than $7041. As the data from this study is now over 10 years old, it is likely that out‐of‐pocket costs have increased due to rising drug prices and increased cost‐sharing requirements from insurers. For example, an estimated 47% of privately insured patients are enrolled in high‐deductible health plans, which require full out‐of‐pocket payments for care until the deductibles are met [26]. In 2025, deductibles for Marketplace silver‐plan enrollees typically exceed $5000 and the percentage of employer‐sponsored insurance covered workers with a deductible of $2000 or more has grown from 26% to 31% [27, 28]. For Medicare beneficiaries, Part B services, such as chemotherapy, requires a 25% copayment for billed services with no out‐of‐pocket cap. Thus, out‐of‐pocket costs of adjuvant chemotherapy could be substantial. Conversely, cost sharing for Medicaid beneficiaries is capped at 5% of family income [29]. Understanding these potential out‐of‐pocket costs attributable to receipt is important when making recurrence score‐guided treatment decisions, as this may affect adherence and outcomes.

In our study, young, low‐risk patients who resided in more affluent neighborhoods had double the probability of receiving adjuvant chemotherapy compared to those living in neighborhoods of high deprivation. We hypothesize younger women living in neighborhoods of low deprivation may choose to pursue overtreatment with adjuvant chemotherapy, even with low risk of recurrence, due to a heightened risk aversion. Compared to older women, younger women diagnosed with breast cancer are at greater risk of having more aggressive tumor biology, a more aggressive disease course, and a poorer prognosis, potentially related to the presence of functional ovaries or reproductive behaviors such as pregnancy and breastfeeding [30, 31]. This risk may contribute to a greater fear of cancer recurrence, which is more common among women with breast cancer who are young, mothers, or who are financially comfortable [32, 33, 34]. Additionally, wealthier women may choose to pursue overtreatment due to a low price sensitivity and greater ability to pay for additional treatment. However, overtreatment may also have unintended consequences of increased risk for chemotherapy‐induced toxicities, decreased quality of life, and increased societal health care costs. Thus, it is important to limit potentially unnecessary overtreatment in low‐risk patients to decrease risk of adverse consequences.

The TAILORx trial found treatment with adjuvant chemotherapy compared to endocrine therapy alone was associated with some survival benefit for women aged ≤ 50 with a medium‐risk recurrence score [17]. However, differences by socioeconomic status were found in our study cohort of younger, medium‐risk patients, with those who resided in impoverished neighborhoods being more likely to receive adjuvant chemotherapy compared to those living in more affluent neighborhoods. This finding may be secondary to the presence of biologically aggressive disease and the ability to tolerate adjuvant treatment. Women living in neighborhoods with less financial resources are more often minoritized and have lower education levels, lower household incomes, and less access to care compared to those living in wealthier neighborhoods [35, 36, 37, 38]. These factors, which often lead to chronic stress and high allostatic load, are also associated with biologically aggressive disease, such as triple negative or inflammatory breast cancers [39, 40]. Oncologists may therefore decide to pursue adjuvant chemotherapy for these patients as a conservative decision‐making approach to mitigate any potential risk of recurrence, especially as these patients are young and potentially better able to tolerate further treatment.

The results of this study should be considered within limitations. The majority of Flatiron data is aggregated from community oncology practices. Therefore, our results may not be generalizable to patients treated by academic practices. Exclusion of patients for missing exposure and/or outcome data may have resulted in potential selection bias, as those excluded were more often Medicaid/other enrollees or treated at a community practice compared to those included in our analyses. Risk of ecological fallacy also exists within our data, as census block‐level indicators of socioeconomic status may not accurately reflect individual‐level socioeconomic status. However, the Yost Index, which is used in our study, is precise to within one decile for 71% of census tracts in the United States and precise to within two deciles for 99% of US census tracks [19]. Another limitation of our study is the potential overestimation of overtreatment among patients aged ≤ 50 with low recurrence scores. The RxPONDER trial demonstrated that premenopausal women with HR + HER2‐eBC with 1–3 positive lymph nodes and an ODX score of 0–25 benefit from the addition of chemotherapy to endocrine therapy, with improvement in invasive disease‐free and distant relapse‐free survival [41]. Since our cohort included stage II patients—some of whom may have had ≤ 3 positive nodes—chemotherapy may have been appropriate in these cases, leading to a possible overestimation of overtreatment in this subgroup. Additionally, our sample of women aged ≤ 50 years is small, which may affect the precision of our results. Guidelines and practice patterns changed over the course of our study. However, we attempted to adjust for these temporal changes by including a covariable for time in all models. The Flatiron dataset uses data predominantly from community practices. Thus, these practices may have different patterns of ODX use to guide treatment when compared to academic medical centers. It is also possible that academic medical centers may use other tests (e.g., Mammaprint, clinical trials) to guide treatment. Additionally, patients who choose to receive care at academic medical centers may be pursuing more aggressive treatment options. We were also unable to capture the potential impact of patient preferences and shared decision‐making during breast cancer treatment, as a considerable number of women at high risk of recurrence in our study did not receive the recommended adjuvant chemotherapy.

## 5 Conclusions

Socioeconomic‐ and insurance‐based inequities, including both overtreatment and undertreatment, were observed in this EHR‐based cohort of women with early‐stage breast cancer eligible for ODX testing. Future research on utilizing personalized medicine approaches without increasing disparities is needed to increase the quality of cancer care delivery, regardless of patients' economic status.

## Author Contributions

Conceptualization: C.P.W., G.R. Methodology: C.P.W., J.S., J.E.D., N.J., A.A., G.R. Analysis: C.P.W., J.S., J.E.D., N.J., A.A., G.R. Writing – original draft: C.P.W., J.S. Writing – review and editing: all authors.

## Funding

This research was supported by a Flatiron Health Equity Grant.

## Conflicts of Interest

Gabrielle B. Rocque—Research: Pfizer, Genentech, Daiichi Sankyo; Consulting: Armada, Gilead, Pfizer. Nusrat Jahan—Consulting: AstraZeneca, Daiichi Sankyo.

## Supporting information


**Table S1:** Comparison of patients included in the analysis vs. excluded due to clinical reasons vs. excluded due to missing values.
**Table S2:** Multivariable models estimating risk of Oncotype DX recurrence score‐guided adjuvant chemotherapy by recurrence risk, neighborhood deprivation, and insurance status: Sensitivity analyses dichotomizing ODX scores into high and low recurrence risk.

## Data Availability

The data that support the findings of this study were originated by and are the property of Flatiron Health Inc. Requests for data sharing by license or by permission for the specific purpose of replicating results in this manuscript can be submitted to #publicationsdataaccess@flatiron.com.
