# Supplementary material for: Socioeconomic‐ and Insurance‐Based Inequities in Oncotype DX Testing and Score‐Guided Treatment
Source: Cancer Med. 2025 Dec 21;14(24):e71485. doi: 10.1002/cam4.71485 (PMC12718550; doi:10.1002/cam4.71485)
Supplement: Supplementary file 1 — Table S1: Comparison of patients included in the analysis vs. excluded due to clinical reasons vs. excluded due to missing values. Table S2: Multivariable models estimating risk of Oncotype DX recurrence score‐guided adjuvant chemotherapy by recurrence risk, neighborhood deprivation, and insurance status: Sensitivity analyses dichotomizing ODX scores into high and low recurrence risk. [file CAM4-14-e71485-s001.docx]

**Supplemental Table 1.** Comparison of patients included in the analysis vs. excluded due to clinical reasons vs. excluded due to missing values.

|  | **Included**  **N=3,814** | **Excluded due to missing values**  **N=2,321** | **Excluded due to clinical reasons**  **N=1,751** |
| --- | --- | --- | --- |
| **Age at diagnosis (mean±SD)** | 62±12 | 65±12 | 62±12 |
| ≤50 years | 669 (18) | 334 (14) | 311 (18) |
| >50 years | 3,145 (82) | 1,987 (86) | 1,440 (82) |
| **Race/ethnicity** |  |  |  |
| Non-Hispanic White | 2,942 (77) | 1,019 (44) | 1,219 (70) |
| BIPOC | 872 (23) | 378 (16) | 532 (30) |
| Unknown | -- | 924 (40) | -- |
| **Neighborhood deprivation** |  |  |  |
| Impoverished | 1,181 (31) | 552 (24) | 587 (34) |
| Affluent | 2,633 (69) | 1,182 (51) | 1,164 (66) |
| Unknown | -- | 587 (25) | -- |
| **Insurance status** |  |  |  |
| Private | 1,792 (47) | 555 (24) | 829 (47) |
| Medicare | 1,365 (36) | 442 (19) | 629 (36) |
| Medicaid/Other* | 657 (17) | 1,324 (57) | 293 (17) |
| **Practice type** |  |  |  |
| Academic | 858 (23) | 170 (7) | 353 (20) |
| Community | 2,956 (78) | 2,151 (93) | 1,398 (80) |
| **Comorbidity count** |  |  |  |
| 0 | 3,527 (92) | 2,201 (95) | 1,677 (96) |
| ≥1 | 287 (8) | 120 (5) | 74 (4) |
| **Cancer stage** |  |  |  |
| I | 2,616 (69) | 1,587 (75) | 1,236 (71) |
| II | 1,033 (27) | 521 (25) | 392 (22) |
| Unknown/Undocumented | 165 (4) | -- | 123 (7) |
| **ECOG** **score** |  |  |  |
| 0 | 2,553 (67) | 1,345 (58) | 1,047 (60) |
| 1 | 726 (19) | 426 (18) | 294 (17) |
| 2 | 98 (3) | 82 (4) | 56 (3) |
| 3 | 24 (1) | 20 (1) | 13 (1) |
| 4 | 5 (0.1) | 3 (0.1) | 0 (0) |
| Unknown | 408 (11) | 445 (19) | 341 (19) |
| **Diagnosis year** |  |  |  |
| 2011 | 169 (4) | 143 (6) | 114 (7) |
| 2012 | 210 (6) | 169 (7) | 124 (7) |
| 2013 | 235 (6) | 185 (8) | 120 (7) |
| 2014 | 291 (8) | 178 (8) | 146 (8) |
| 2015 | 317 (8) | 192 (8) | 143 (8) |
| 2016 | 351 (9) | 172 (7) | 139 (8) |
| 2017 | 339 (9) | 185 (8) | 140 (8) |
| 2018 | 389 (10) | 185 (8) | 148 (8) |
| 2019 | 392 (10) | 199 (9) | 139 (8) |
| 2020 | 359 (9) | 163 (7) | 106 (6) |
| 2021 | 303 (8) | 205 (9) | 147 (8) |
| 2022 | 307 (8) | 195 (8) | 150 (9) |
| 2023 | 152 (4) | 150 (6) | 135 (8) |

*Other Government Program, Other Payer - Type Unknown, Workers Compensation

**Supplemental Table 2.** Multivariable models estimating risk of Oncotype DX recurrence score-guided adjuvant chemotherapy by recurrence risk, neighborhood deprivation, and insurance status: Sensitivity analyses dichotomizing ODX scores into high and low recurrence risk.

|  | **Women aged ≤50 years who received ODX testing**  **n=314** |
| --- | --- |
|  | **RR (95% CI)** |
| **Neighborhood Deprivation** | |
| Low risk, affluent neighborhood | Ref. |
| Low risk, impoverished neighborhood | 1.17 (0.49-2.80) |
| High risk, affluent neighborhood | Ref. |
| High risk, impoverished neighborhood | 1.02 (0.73-1.42) |
| **Insurance Status** | |
| Low risk, privately insured | Ref. |
| Low risk, publicly/other insured | 1.23 (0.51-2.94) |
| Low risk, Medicare beneficiary | -- |
| Low risk, Medicaid/other beneficiary | -- |
| High risk, privately insured | Ref. |
| High risk, publicly/other insured | 0.88 (0.61-1.26) |
| High risk, Medicare beneficiary | -- |
| High risk, Medicaid/other beneficiary | -- |
